# Novel small scale TFF cell retention device for perfusion cell culture systems

**DOI:** 10.1186/1753-6561-9-S9-P25

**Published:** 2015-12-14

**Authors:** Chris S Martin, Jorge Padilla-Zamudio, Doug Rank, Patrick McInnis, Mikhail Kozlov, Sarah Reynolds, Joe Parella, Lee Madrid

**Affiliations:** 1 EMD Millipore Corporation, Bedford, MA, 01730, USA

## Background

Perfusion processes have traditionally been used for the generation of unstable proteins in cell culture systems. The use of perfusion for production of stable proteins has been limited by low product concentration, media costs, and system complexity. With the advent of new single-use technology and high producing cell lines, perfusion processes are gaining increased attention from industry. Also, the enhanced productivity of perfusion bioreactor compared to fedbatch enables use of smaller single-use systems, both for clinical scale production as well as potentially for manufacturing scale. These new perfusion processes cannot only be used as a production platform but also for process intensification of fed-batch processes. Perfusion can be used to dramatically increase the cell density of the N-1 bioreactor to accelerate the production process and achieve gains in efficiency.

The critical components of a perfusion system are the bioreactor controller, cell culture vessel, and cell retention device. The cell retention device or system is often used to define the type of perfusion system with gravity-based and filtration devices being the most common.

This study will show the application of a small-scale tangential flow microfiltration device(prototype small scale Prostak™) in a perfusion test platform. This prototype device was derived from EMD Millipore Prostak™microfiltration family of products typically used in the primary clarification of cell culture.

## Materials and Methods

The 3 channel ProstakTM MF 0.2 um 640 cm2 membrane prototype small scale devices were obtained from EMD Millipore Process Solutions R&D. The devices were pre-sterilized by autoclave. The source and definition of the components of the bioreactor and system control set up are listed (Table 1). The recombinant protein producing CHOS cell line used in this study was previously shown to express adequately over time and grow rapidly using a shake flask semicontinous perfusion model.

**Table 1:** Perfusion Process Details.

Materials and Equipment	
Cell Line	rCHOS

Vessel and Controller	3L Applikon^® ^Glass Bioreactor Finesse^® ^TruBio^™^DeltaV
Additional Equipment	Single-use recirculation Levitronix™ Pump, Single-use Mobius^® ^20L bags (Harvest and Medium)
Cell Growth Monitoring - two methods	Vi-CELL^® ^(Trypan blue exclusion method)
	Biomass by capacitance (iBiomass™ - Fogale™)
Bioreactor Conditions	
Vessel Temp (C)	37.0
pH Set Point	7.0 ( ± 0.2)
Dissolved Oxygen (DO)	50%
Aeration Gas/Location	Air-Oxygen / Open Pipe
Inoculation Density	3~5E5 vc/mL
Inoculation Agitation	250 rpm (30W/m^3^)
Working Volume (L)	1.5 L
Daily Cell Bleed	Manually (w/ peristaltic pump)

## Results

**Figure 1:**
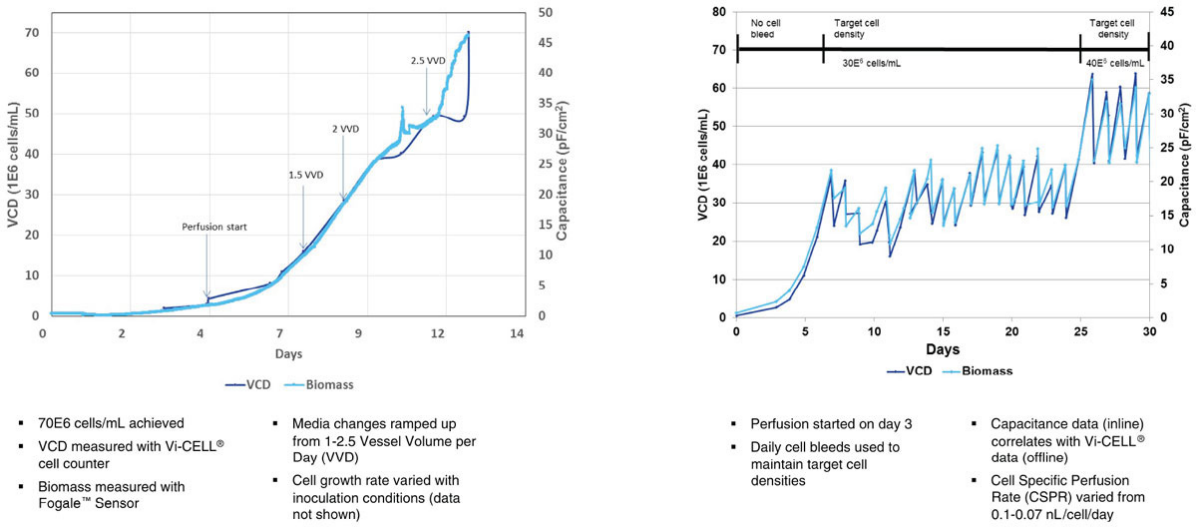
**Results from two separate bioreactor perfusion runs using the ssProstak™**.

Results from two separate ssProstakTM perfusion runs are shown (Figure 1). In the first graph, we show the ability to grow our model cells to a density of 70E6 cells/ml in 12 days. These high cell densities would be enabling in N-1 bioreactor applications to shorten seed train and overall production times. Additionally, the FogaleTM biomass sensor is confirmed as an accurate method to track cell viability on-line. In the second graph, a longer perfusion run is achieved by utilizing manual daily cell bleed. In the initial perfusion phase (days 5-24) the cell density is maintained at 30E6 cells/ml. Healthy cell growth is achieved during this time period as evidenced by the cell growth curve after each bleed. In the later perfusion phase, starting at day 25, cell density was maintained at 40E6 cells/ml with similar cell growth characteristics and a final viability of >85%. The utility of using a biomass sensor was also shown in this run, and we could envision that the sensor data could be used to automate cell bleeding or the media perfusion rate. This 30 day perfusion process with the ssProstakTM is an example of how ProstakTM could be used in a production perfusion process where the protein of interest was continuously harvested from the permeate.

## Conclusions

We have demonstrated that a new small scale TFF device (ssProstakTM) can be used in perfusion applications. Currently bioreactor perfusion processes are used for number reasons including process intensification and continuous upstream. The data suggest the utility of ProstakTM in accomplishing these process goals. The performance metrics measured in this study are similar to those obtained with other perfusion processes using hollow fiber TFF cell retention devices, either normal flow or alternating.

